# Ionizing Radiation Potentiates Dihydroartemisinin-Induced Apoptosis of A549 Cells via a Caspase-8-Dependent Pathway

**DOI:** 10.1371/journal.pone.0059827

**Published:** 2013-03-25

**Authors:** Tongsheng Chen, Min Chen, Jingqin Chen

**Affiliations:** MOE Key Laboratory of Laser Life Science and SATCM Third Grade Laboratory of Chinese Medicine and Photonics Technology, College of Biophotonics, South China Normal University, Guangzhou, China; Temple University, United States of America

## Abstract

This report is designed to explore the molecular mechanism by which dihydroartemisinin (DHA) and ionizing radiation (IR) induce apoptosis in human lung adenocarcinoma A549 cells. DHA treatment induced a concentration- and time-dependent reactive oxygen species (ROS)-mediated cell death with typical apoptotic characteristics such as breakdown of mitochondrial membrane potential (Δψ_m_), caspases activation, DNA fragmentation and phosphatidylserine (PS) externalization. Inhibition of caspase-8 or -9 significantly blocked DHA-induced decrease of cell viability and activation of caspase-3, suggesting the dominant roles of caspase-8 and -9 in DHA-induced apoptosis. Silencing of proapoptotic protein Bax but not Bak significantly inhibited DHA-induced apoptosis in which Bax but not Bak was activated. In contrast to DHA treatment, low-dose (2 or 4 Gy) IR induced a long-playing generation of ROS. Interestingly, IR treatment for 24 h induced G2/M cell cycle arrest that disappeared at 36 h after treatment. More importantly, IR synergistically potentiated DHA-induced generation of ROS, activation of caspase-8 and -3, irreparable G_2_/M arrest and apoptosis, but did not enhance DHA-induced loss of Δψ_m_ and activation of caspase-9. Taken together, our results strongly demonstrate the remarkable synergistic efficacy of combination treatment with DHA and low-dose IR for A549 cells in which IR potentiates DHA-induced apoptosis largely by enhancing the caspase-8-mediated extrinsic pathway.

## Introduction

Lung cancer is the leading cause of cancer related deaths worldwide in recent years [1]. It has been estimated that more than one million people die of it annually and over 1.4 millions are diagnosed every year [2]. Ionizing radiation (IR) therapy is widely considered as an effective therapeutic method for the control of lung cancer in the clinical applications, and considerably improves the length of survival for patients [3]. In response to IR, cells may undergo either cell cycle arrest and DNA damage repair or even apoptosis if the damage is beyond repaired [4], [5]. The major mechanism involves the increase of DNA damage and decrease of DNA mismatch repair, in which reactive oxygen species (ROS) may modulate the signaling pathway through a wide variety of responses. Furthermore, some investigations demonstrate that, after IR treatment, transcription factors such as NF-κB and p53 upregulate the antiapoptotic proteins Bcl-2 and Bcl-x_L_ through the intrinsic apoptosis pathway [6]. Although it is the most effective modality in cancer treatment, IR, especially the high-dose IR, also provokes lots of side effects on normal tissues such as undesirable inflammatory reactions [6], [7]. Therefore, natural products with lower side effects combined with low-dose IR have received increasing attention in recent years for the discovery of novel cancer therapeutic methods.

Artemisinin (ART), a natural product isolated from Chinese medicinal herb Artemisia annua L. (qinghao), and its derivatives (ARTs) such as dihydroartemisinin (DHA) and artesunate (ARTE) have been shown to have anticancer effects by induction of apoptosis without obvious side effects [8]–[10]. ROS from the reaction of endoperoxide bridge in ARTs plays important roles in ARTs-induced apoptosis [11]–[14]. DHA is widely considered as one of the most effective ARTs in terms of apoptosis induction, although the detailed mechanisms are still needed to be further elucidated [15], [16]. Many recent studies, including these from our laboratory, have shown that cell apoptosis induced by DHA is implicated in the extrinsic (death receptor) and intrinsic (mitochondrial) apoptosis pathways or either of them, which may be companied by the generation of ROS, activation of caspases and decrease of Bcl-2/Bax expression ratio [17]–[19]. Specifically, Handrick et al (2010) [20] have demonstrated that DHA induces apoptosis by a Bak-dependent intrinsic pathway without the activation of caspase-8 in Jurkat cells. It has been reported that DHA treatment alone or in combination with other therapies such as gemcitabine activates transcription factor NF-κB or p53 to start intrinsic apoptosis pathway *in vitro* and *in vivo* [15], [17]. Our recent studies have demonstrated that caspase-8, -9 and -3 play important roles in DHA-induced apoptosis of human lung adenocarcinoma ASTC-a-1 cells [18], [19].

This report is designed to explore the molecular mechanism by which low-dose IR enhances DHA-induced apoptosis in another human lung adenocarcinoma A549 cells and provide a molecular basis for the use of combination therapy with DHA and IR to human lung adenocarcinoma. Our data show for the first time that low-dose IR remarkably potentiates the cytotoxic action of DHA in a synergistic manner through a caspase-8-dependent apoptosis pathway.

## Results

### DHA Induces Apoptosis of A549 Cells

**Figure 1:**
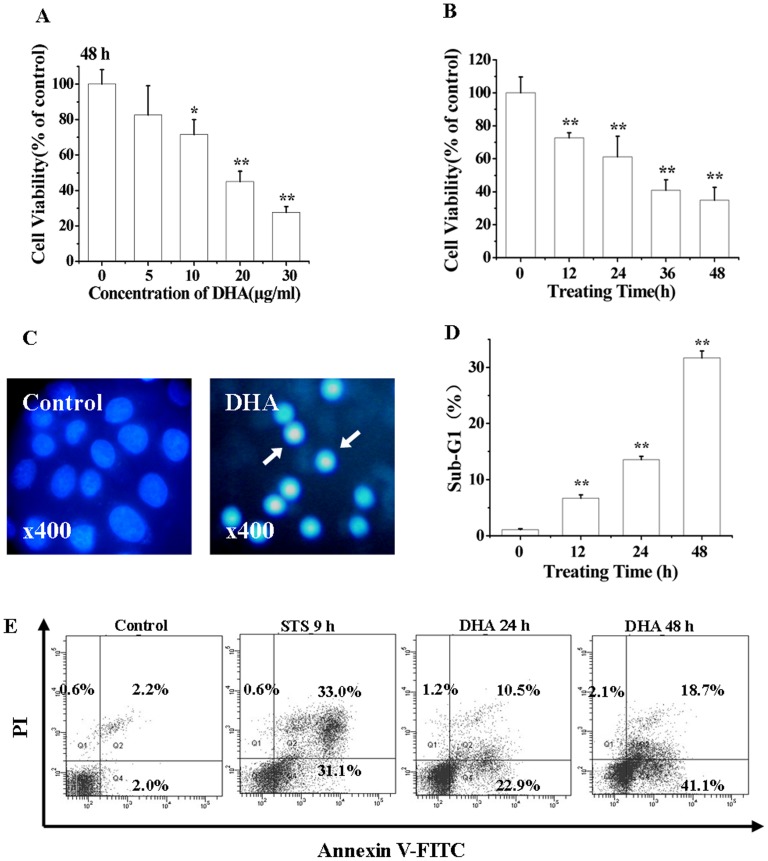
DHA induces apoptosis. (A) DHA-induced concentration-dependent reduction of cell viability assessed by CCK-8 assay. Cells were incubated with various concentrations of DHA (0, 5, 10, 20, 30 µg/ml) for 48 h, *P<0.05, compared with control; **P<0.01, compared with control. (B) DHA-induced time-dependent reduction of cell viability by CCK-8 assay. Cells were incubated with 20 µg/ml of DHA for different time (0, 12, 24, 36, 48 h). **P<0.01, compared with control. (C) DHA-induced nuclear condensation of cells stained by Hoechst 33258 after treatment for 24 h. Images were recorded using a digital camera with 1280×1280 pixels resolution. Magnification 400. (D) DHA-induced Sub-G_1_ cell cycle arrest. Cells were cultured with 20 µg/ml of DHA for 0, 12, 24 or 48 h and then stained with 5 µg/ml of PI before being analyzed by FCM. (E) DHA-induced apoptosis assessed by FCM analysis. Cells were treated with 20 µg/ml of DHA for 0, 24 and 48 h, and then analyzed by FCM after staining with Annexin V-FITC/PI.

We first characterized the cytotoxic action of DHA to A549 cells. After exposure of the cells to different concentrations of DHA (0–30 µg/ml) for 48 h (Fig. 1A) and to 20 µg/ml of DHA for different time (0–48 h), the Cell Counting Kit-8 (CCK-8) assay was used to assess the cell viability. Our data demonstrated that DHA effectively induced concentration- and time-dependent cytotoxicity (Figs. 1A and 1B). After treatment with 10, 20, or 30 µg/ml of DHA, microscopic imaging of living cells stained with propidium iodide (PI) showed that the treatment using 30 µg/ml of DHA induced significantly necrosis (data not shown), while the treatment using 20 µg/ml of DHA induced the maximum level of apoptosis. Therefore, the concentration of 20 µg/ml of DHA was adopted in the following experiments.

To determine whether DHA induced cell death in apoptosis fashion, Hoechst 33258, PI staining and Annexin V/PI staining assay were used to examine the characteristics of apoptosis. Microscopic imaging of living cells stained with Hoechst 33258 showed apoptosis-related chromatin condensation and margination in DHA-treated cells (24 h) (Fig. 1C). Flow cytometry (FCM) analysis of the DNA content of cells stained with PI demonstrated that the proportion of cells with Sub-G_1_ DNA content indicative of apoptotic cells increased from about 1.0% (control) to 6.5%, 13.9% and 32.6% at 12, 24 and 48 h after DHA treatment (Fig. 1D). Annexin V/PI staining assay was used to examine the integrity of cell membrane and the externalization of phosphatidylserine (PS), the typical characteristics of apoptosis. Staurosporine (STS)-treated cells were used as positive control. DHA treatment induced a marked increase in the percentage of cells with PS externalization and loss of the cell membrane integrity from 4.2% (control) to 33.4% (24 h) and 59.8% (48 h) (Fig. 1E), further demonstrating the notion that DHA treatment induced a time-dependent apoptosis.

### Low-dose IR Dramatically Enhances DHA-elicited ROS Production

**Figure 2:**
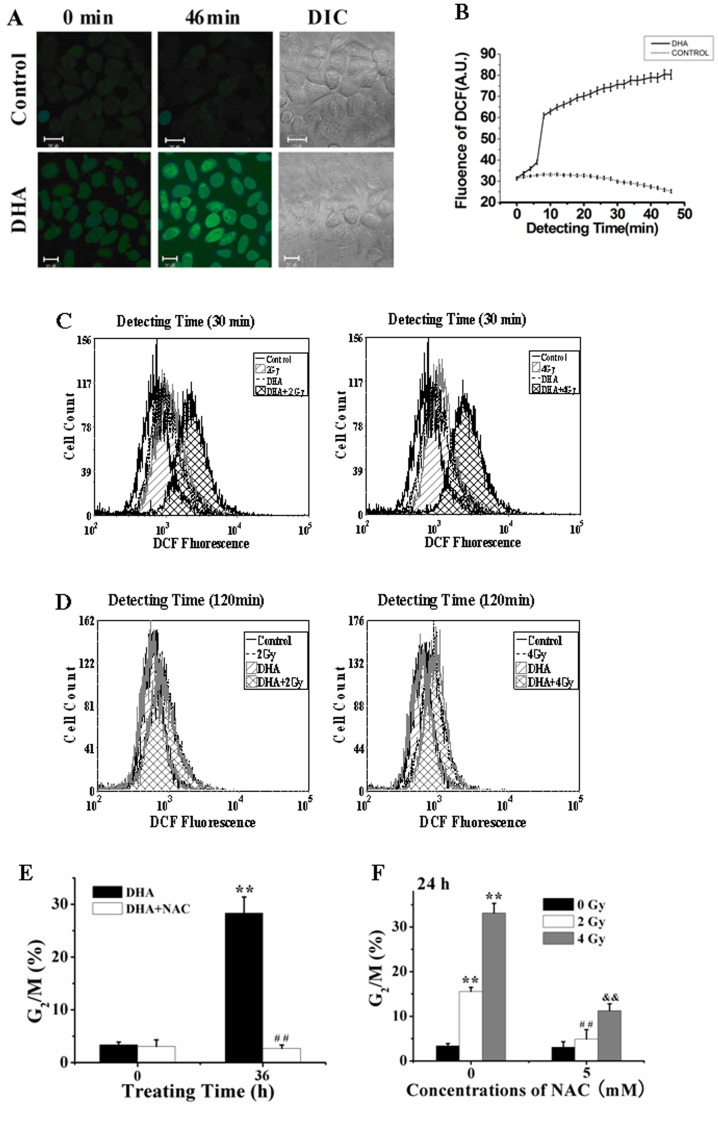
ROS-dependent G2/M cell cycle arrest by DHA and IR respectively. (A) Dynamical fluorescence images of ROS generation in living cells after DHA treatment. Cells were incubated with 20 µM DCFH-DA, an oxidation-sensitive fluorescent probe, for 30 min in the dark and then treated with DHA. The levels of intracellular ROS were monitored by a confocal microscope. Scale bar: 20 µm. (B) Dynamics of DHA-induced ROS generation corresponding to Figure 2 (A). (C and D) FCM assay of ROS generation at 30 min (C) and 120 min (D) after IR, DHA and combination treatment, respectively. (E and F) ROS-dependent G_2_/M arrest induced by IR (E) and DHA (F) respectively analyzed by FCM. Cells were irradiated with IR or DHA in the presence or absence of NAC, and then stained with 5 µg/ml of PI before being analyzed by FCM. **P<0.01, compared with control; ^##^ *P*<0.01, compared with DHA treatment alone (E) and ^##^ *P<0.01* and ^&&^ *P<0.01*, compared with IR treatment alone (F).

Confocal imaging of living cells incubated with 20 µM 2′,7′-Dichlorodihydrofluorescein diacetate (DCFH-DA), an oxidation-sensitive fluorescent probe, showed that DHA treatment induced a rapid generation of ROS within 10 min (Figs. 2A and 2B). As shown in Figure 2C, treatment with DHA and low-dose IR (4 Gy) respectively induced a considerable ROS production, and IR treatment remarkably enhanced DHA-elicited ROS production at 30 min after treatment in a synergistic manner. However, we only detected the IR-induced ROS at 120 min after treatment (Fig. 2D), indicating that ROS elicited rapidly from DHA leaked out the cells, whereas IR treatment induced a long-playing generation of ROS.

FCM analysis showed that treatment with DHA and IR respectively induced a significant G2/M cell cycle arrest that was remarkably attenuated by N-acetyl cysteine (NAC) (one of scavengers of ROS) pretreatment (Figs. 2E and 2F), implying that ROS played important roles in both IR- and DHA-induced G_2_/M arrest.

### Both the Intrinsic and Extrinsic Apoptosis Pathways Play Important Roles in DHA-induced Apoptosis

**Figure 3:**
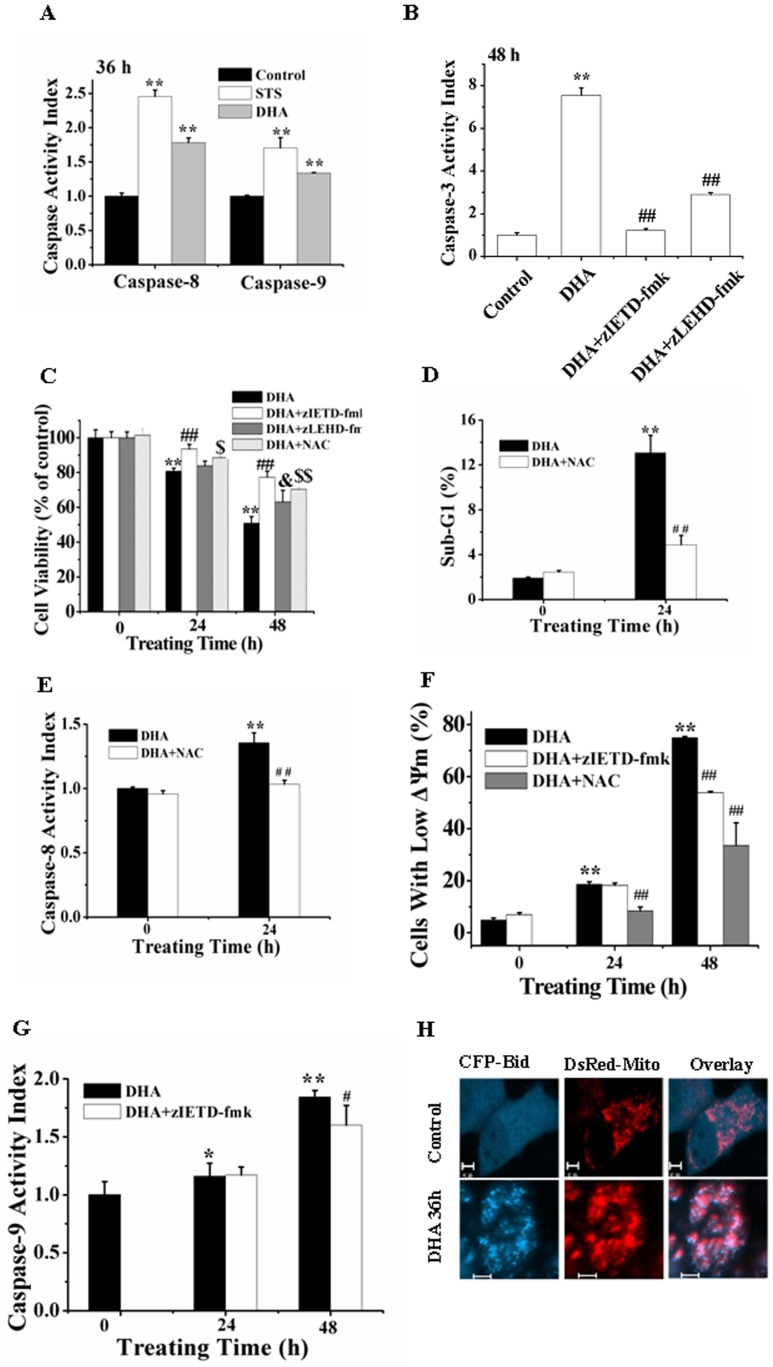
DHA induces apoptosis via both extrinsic and intrinsic apoptosis pathways. (A) DHA induced activation of caspase-8 and -9 assessed by fluorometric assay. Cells were treated with DHA for 36 h. **P<0.01, compared with control. (B) DHA induced caspase-8- and -9-dependent caspase-3 activation by fluorometric assay. Cells were treated with DHA for 48 h in the presence or absence of zIETD-fmk and zLEHD-fmk, respectively. **P<0.01, compared with control; *^##^P*<0.01, compared with DHA treatment alone. (C) DHA induced caspase-8- and -9-dependent cytotoxicity assessed by CCK-8 assay. Cells were treated with DHA for 24 and 48 h in the presence or absence of zIETD-fmk and zLEHD-fmk, respectively. *8*P<0.01*, compared with control; ^$^ *P<0.05*, ^$$^ *P<0.01* and ^&^ *P*<0.05, compared with DHA treatment alone. (D) DHA ROS-mediated apoptosis assessed by FCM. **P<0.01, compared with control; ^##^ *P<0.01*compared with DHA alone. (E) DHA induced ROS-dependent caspase-8 activation. **P<0.01, compared with control; ^##^ *P<0.01*, compared with DHA treatment alone. (F) DHA induced ROS- and caspase-8-dependnent loss of Δψ_m_ determined by FCM analysis. **P<0.01, compared with control; ^##^ *P<0.01*, compared with DHA treatment alone. (G) DHA induced caspase-8-dependent caspase-9 activation. *P<0.05 and **P<0.01, compared with control; ^#^ *P<0.05*, compared with DHA treatment alone. (H) Typical fluorescence images of Bid translocation to mitochondria inside single living cell after DHA treatment for 36 h. Control cells show the uniform distribution of Bid, while DHA-treated cells show the co-localization between Bid and mitochondria. Scale Bar: 5 µm.

To verify whether caspase-8 and -9 are involved in DHA-induced apoptosis, fluorometric substrate assay was used to detect caspases activity. STS-treated cells were used as positive control. DHA treatment for 36 h substantially induced activation of caspase-8, -9 and -3 (Figs. 3A and 3B), and pretreatment with zIETD-fmk or zLEHD-fmk, the inhibitors of caspase-8 or -9, dramatically prevented the DHA-induced caspase-3 activation (Fig. 3B), indicating that caspase-8 and -9 acted as the upstream regulators of casapse-3 activation. CCK-8 assay showed that inhibition of caspase-8 or -9 by pretreatment with zIETD-fmk or zLEHD-fmk significantly prevented the cytotoxicity of DHA treatment for 48 h (Fig. 3C), demonstrating the important roles of caspase-8 and -9 in DHA-induced apoptosis. Moreover, compared with zLEHD-fmk, zIETD-fmk pretreatment led to a more potent prevention on the cytotoxicity of DHA (Fig. 3C).

We also found that NAC pretreatment potently prevented the cytotoxicity of DHA (Fig. 3C), indicating that ROS played an important role in DHA-induced apoptosis, which was further verified by the prevention of NAC pretreatment on DHA-induced Sub-G_1_ cell cycle arrest (Fig. 3D). Moreover, NAC pretreatment remarkably prevented DHA-induced activation of caspase-8 (Fig. 3E) and loss of the mitochondrial membrane potential (Δψ_m_) (Fig. 3F), indicating that ROS elicited from DHA mediated the intrinsic and extrinsic apoptosis pathways. Moreover, zIETD-fmk pretreatment significantly inhibited the DHA-induced loss of Δψ_m_ (Fig. 3F) and activation of caspase-9 (Fig. 3G), demonstrating that the extrinsic pathway participated in enhancing the intrinsic pathway to accelerate DHA-induced apoptosis. To evaluate whether BH3-only protein Bid bridges the extrinsic and intrinsic pathway, we used confocal imaging to monitor the spatial distribution of Bid inside living cells co-expressing CFP-Bid and DsRed-Mito, and found that DHA treatment for 36 h induced a significant translocation of tBid to mitochondria (Fig. 3H), indicating that caspase-8 cleaved Bid to tBid that translocated to mitochondria to potentiate the intrinsic apoptosis pathway.

### Bax and Bcl-x_L_ are Involved in DHA-induced Apoptosis

**Figure 4:**
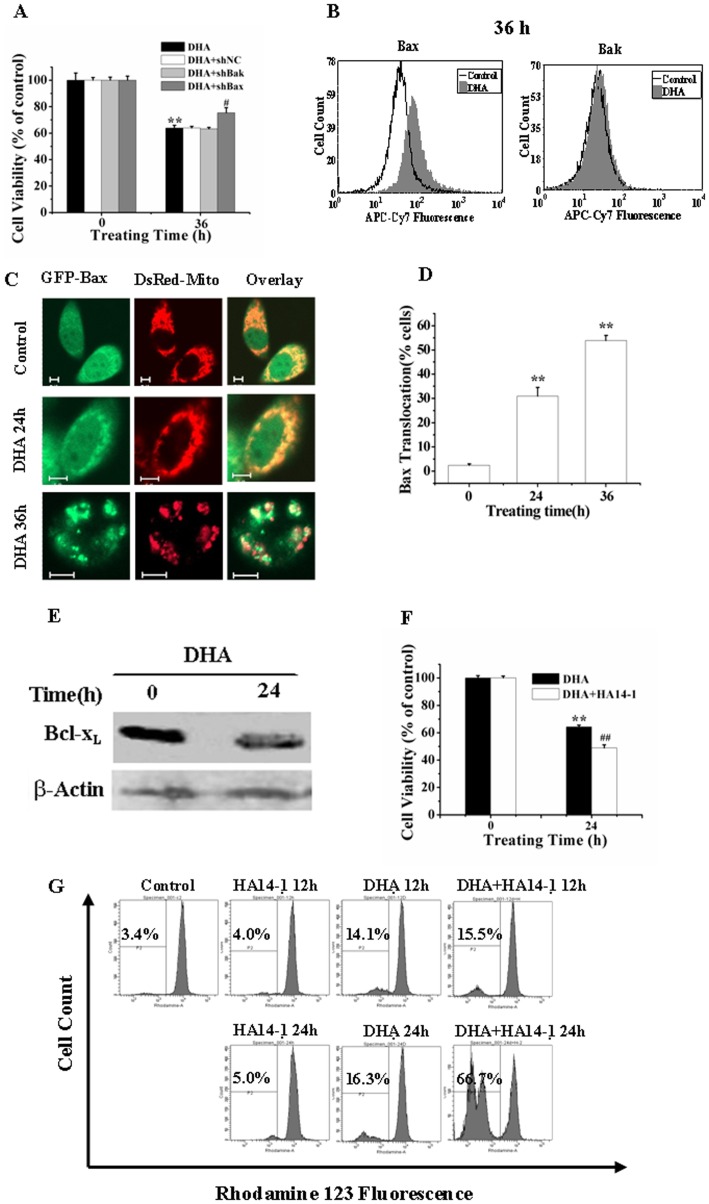
Bax and Bcl-x_L_ are involved in the DHA-induced apoptosis. (A) Silencing of Bax but not Bak prevented DHA-induced decreasing of cell ability assessed by CCK-8. Cells with shBax or shBak were treated with DHA for 36 h. ShNC was a negative control for shRNA. **P<0.01, compared with control; ^#^ *P<0.05*, compared with DHA treatment alone. (B) DHA induced the activation of Bax but not Bak analyzed by FCM analysis. (C) Typical fluorescence images of cells showing Bax translocation to mitochondria inside single living cell after DHA treatment for 24 h and 36 h. Scale Bar: 5 µm. (D) Quantification of cells showing GFP-Bax translocation from about 200 cells per treatment in 15 to 20 randomly selected image frames from three independent experiments. **P<0.01, compared with control. (E) DHA induced a decrease expression of Bcl-x_L_ assessed by Western blot analysis. Bcl-x_L_ expressions in control, DHA-treated cells were detected by Western blot using antibodies against Bcl-x_L_ and β-actin. (F) HA14-1 pretreatment enhanced DHA-induced cytotoxicity. Cells were cultured with DHA for 0 or 36 h with or without the addition of 10 µM HA14-1. **P<0.01, compared with control; *^##^P*<0.01, compared with DHA treatment alone. (G) HA14-1 accelerated DHA-induced loss of Δψ_m_. Cells were cultured with DHA for 0, 12 and 24 h in the presence or absence of 10 µM HA14-1, and stained with 1 µM Rho123 before being analyzed by FCM.

To further substantiate the detailed molecular mechanism of the intrinsic apoptosis pathway, we determined the roles of Bax and Bak during DHA-induced apoptosis by using RNA Interference (RNAi). Western blotting analysis demonstrated that shBax- or shBak-transfected cells showed a significant down-regulation of the expression of Bax or Bak (data not shown). CCK-8 assay showed that silencing of Bax but not Bak significantly attenuated the decrease of cell viability after DHA treatment for 36 h (Fig. 4A), demonstrating that Bax rather than Bak was involved in the DHA-induced apoptosis. Bax/Bak activation experiences an N-terminal conformational transform which can be measured by antibodies that specifically recognize the active conformer of Bax (6A7) and Bak (Ab-2). FCM analysis showed that DHA treatment for 36 h induced a marked activation of Bax but not Bak (Fig. 4B). Confocal fluorescence microscopy imaging of single living cells co-expressing GFP-Bax and DsRed-Mito showed that GFP-Bax co-localized with DsRed-Mito in response to DHA treatment for 24 and 36 h (Fig. 4C), and statistical results from 200 cells in three independent experiments showed that the percentage of cells showing Bax translocation increased from 2.59% (control) to 30.95% for 24 h and to 53.95% for 36 h after DHA treatment (Fig. 4D), further demonstrating the important roles of Bax in DHA-induced apoptosis.

It has been demonstrated that some of the antiapoptotic members of Bcl-2 family could inhibit the activation of Bax during apoptosis [21]. Here, we wonder whether Bcl-x_L_, a strong antiapoptotic member of Bcl-2 family, was implicated in DHA-induced apoptosis. Western blotting analysis demonstrated that DHA treatment markedly declined the expression level of Bcl-x_L_ (Fig. 4E), and pretreatment with HA14-1, an inhibitor of Bcl-x_L_, significantly enhanced the DHA-induced cytotoxicity (Fig. 4F) and loss of Δψ_m_ (Fig. 4G), suggesting that Bcl-x_L_ participated in DHA-induced apoptosis.

Collectively, our data demonstrate that Bax and Bcl-x_L_ act as upstream regulators to trigger the intrinsic pathway in DHA-induced apoptosis.

### IR Enhances DHA-induced G_2_/M Arrest and Apoptosis

**Figure 5:**
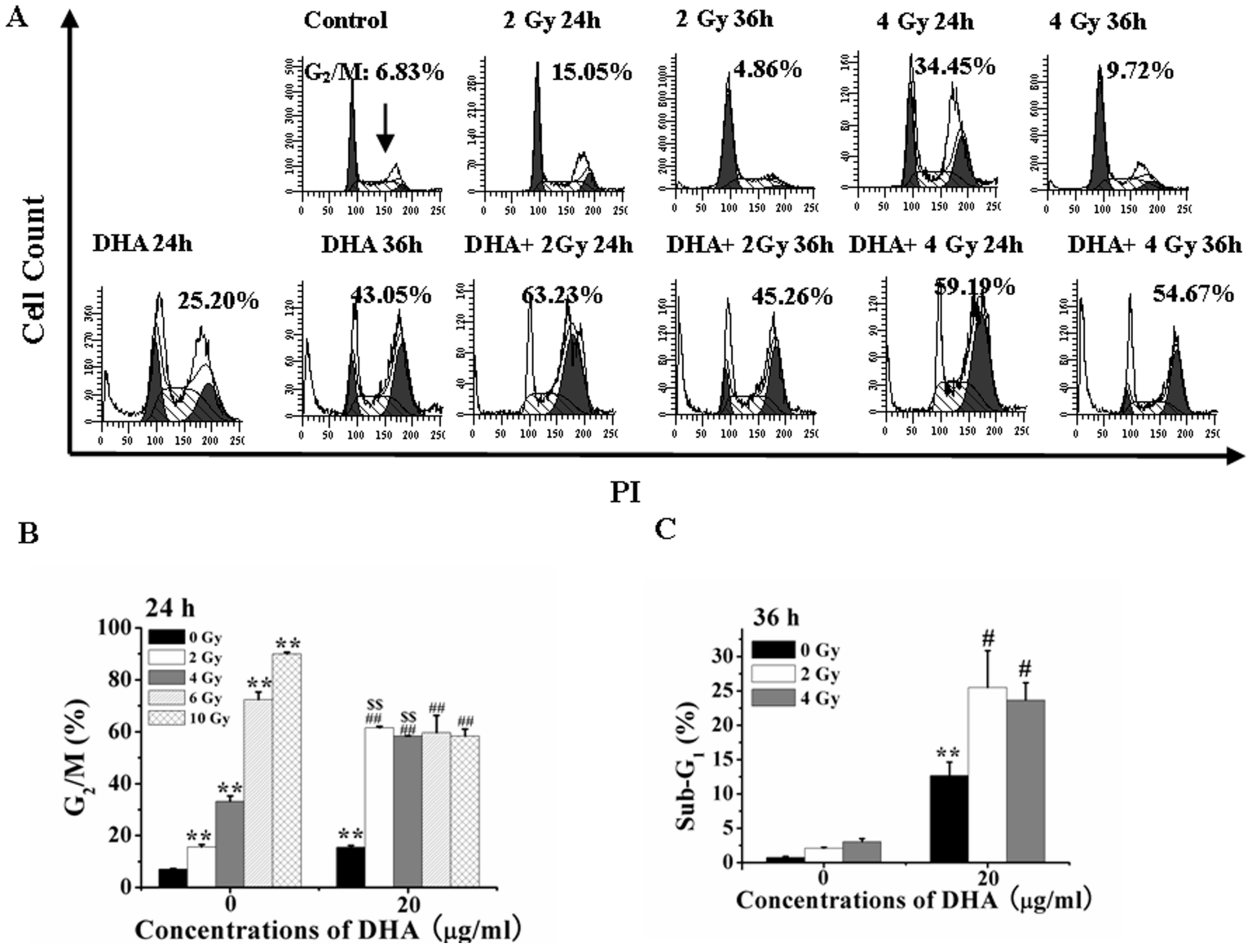
IR synergistically enhances DHA-induced G_2_/M arrest and apoptosis. (A) FCM analysis of cells cycle after low-dose IR treatment for 24 h and 36 h in the presence or absence of DHA. (B and C): IR potentiated the DHA-induced G_2_/M arrest at 24 h (B) and apoptosis at 36 h (C) analyzed by FCM. **P<0.01, compared with treatment with control; *^##^P*<0.01, compared with DHA treatment alone, *^$$^P*<0.01 compared with 2 Gy IR treatment; *^&&^P*<0.01, compared with 4 Gy IR treatment. Cells treated with different doses of IR were cultured with 20 µg/ml of DHA for indicated time and then stained with 5 µg/ml of PI before being analyzed by FCM.

FCM was used to evaluate the effects of IR treatment, DHA treatment and combination treatment with both IR and DHA on cell cycle arrest. Figure 5A showed a typical FCM analysis, and Figures 5 B and 5C showed the statistical results from three independent experiments. As shown in Figure 5A, the cells at 24 h after treatment with low-dose (2 and 4 Gy) IR showed a significant G_2_/M cell cycle arrest that, however, disappeared at 36 h after treatment. Moreover, the IR treatment did not induce Sub-G1 cell cycle arrest, indicating that IR treatment did not induce apoptosis (Fig. 5A). DHA treatment for 24 and 36 h significantly induced a time-dependent irreversible G_2_/M and Sub-G1 cell cycle arrest (Fig. 5A, low panel). Moreover, compared with DHA treatment alone, combination treatment with DHA and low-dose (2 and 4 Gy) IR showed a synergistic increase of G_2_/M and Sub-G1 cell cycle arrest (Fig. 5A, low panel). From Figure 5B, we found that IR treatment significantly induced a dose (0–10 Gy)-dependent G_2_/M cell cycle arrest, and that DHA co-treatment remarkably enhanced the 2 and 4 Gy of IR-induced G_2_/M cell cycle arrest, but significantly decreased the 6 and 10 Gy of IR-induced G_2_/M cell cycle arrest, indicating the synergistic action of the combination treatment with DHA and low-dose (2 and 4 Gy) IR in G_2_/M cell arrest. Moreover, we also found that low-dose IR (2 and 4 Gy) remarkably enhanced DHA-induced Sub-G_1_ arrest (Fig. 5C), further indicating the synergistic action of the combination treatment in apoptosis.

### The Extrinsic Apoptosis Pathway Plays Key Roles in the Synergistic Action of the Combination Treatment with IR and DHA

**Figure 6:**
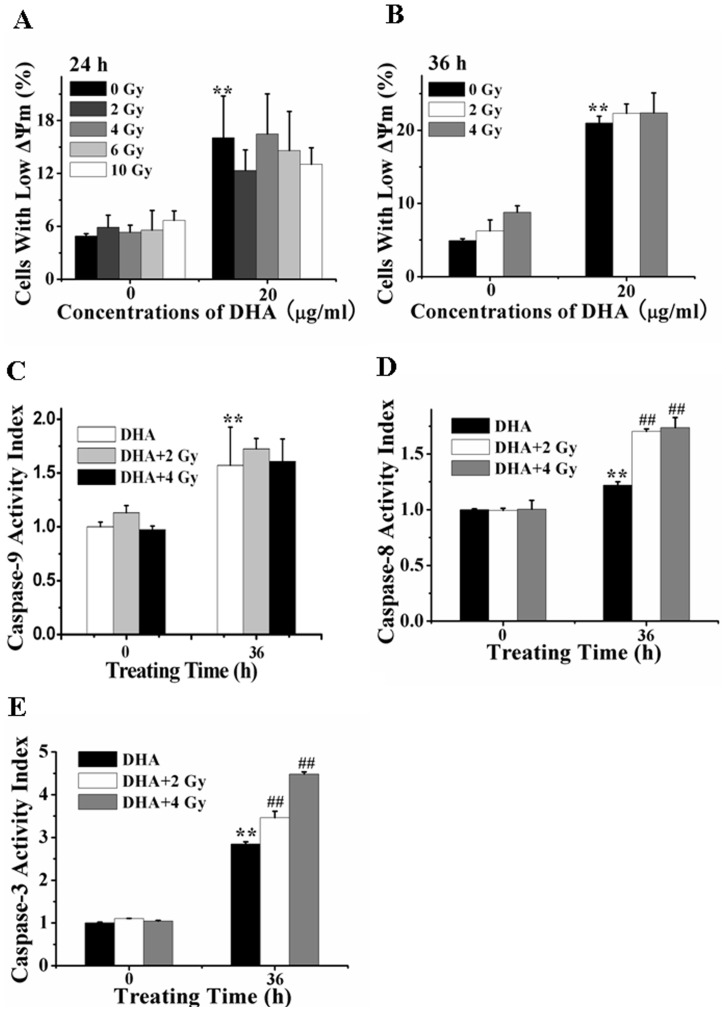
IR potentiates DHA-induced extrinsic apoptosis pathway. (A–B): IR did not accelerate the DHA-induced loss of Δψ_m_ at 24 h (A) and 36 h (B) after treatment assessed by FCM. **P<0.01, compared with control. (C) IR did not accelerate DHA-induced caspase-9 activation. **P<0.01, compared with control. (D and E) IR accelerated DHA-induced activation of caspase-8 (D) and -3 (E). Cells treated with IR were then cultured with DHA for 36 h. Caspase-8, -9 and -3 activities were measured by the fluorescence substrate Ac-IETD-AFC, Ac-LEHD-AFC and Ac-DEVD-AFC, respectively. **P<0.01, compared with control, *^##^P*<0.01, compared with treatment with DHA alone.

To investigate whether the intrinsic apoptosis pathway is involved in the synergistic effect of the combination treatment with low-dose IR and DHA, FCM analysis was firstly used to assess the effect of IR treatment on DHA-induced loss of Δψ_m_. We found that IR (2–10 Gy) treatment alone did not induced significant loss of Δψ_m_ compared with control (Figs. 6 A and 6B), and IR (2–10 Gy) treatment also did not potentiate the loss of Δψ_m_ induced by DHA treatment for 24 and 36 h (Figs. 6A and 6B), suggesting that the intrinsic apoptosis pathway was not involved in the synergistic effect of the combination treatment, which was further verified by the data that IR treatment did not increase the DHA-induced activation level of caspase-9 (Fig. 6C). However, fluorometric substrate assay showed that low-dose (2 or 4Gy) IR substantially enhanced the DHA-induced activation of caspase-8 and -3 (Figs. 6D and 6E), suggesting the important role of caspase-8 and -3 in the synergistic cytotoxicity of the combination treatment. Collectively, these data implicate that the extrinsic apoptosis pathway plays key roles in the synergistic action of the combination treatment with DHA and low-dose IR for A549 cells.

## Discussion

Recent studies suggest a potential use of DHA as an anticancer agent [15], [18] and combination treatment with DHA and low-dose IR as a promising therapeutic strategy of cancer therapy [20]. However, the detailed molecular mechanisms by which combination treatment induces apoptosis remain unclear [12], [20]. Here, we show that DHA induces apoptosis of A549 cells via the extrinsic and intrinsic apoptosis pathways. Moreover, we demonstrate for the first time that low-dose (2 or 4 Gy) IR potentiates DHA-induced apoptosis in a synergistic fashion in which the extrinsic apoptosis pathway plays a key role.

Of note, treatment with IR alone seems to work better than with DHA on G2/M cell cycle arrest (Fig. 5B). Cells in G2/M arrest decreased from about 90% induced by treatment with 10 Gy IR alone to about 60% induced by the combination treatment with IR and DHA (Fig. 5B). It may be due to the DHA-induced cell death which changes cell cycle from G2/M phase to Sub-G1 phase. In combination with our data that compared with DHA treatment alone, the combination treatment does not significantly enhance the decrease of mitochondrial membrane potential (Figs 6A and 6B) and the activation of caspase-9(Fig. 6C). We infer that the intrinsic pathway does not play a significant role in the synergistic action of the combination treatment. In addition, compared with 2Gy, 4–10 Gy of IR treatment did not significantly enhance the DHA-induced G2/M arrest, Sub-G1 arrest (Figs. 5B and 5C) or decrease of mitochondrial membrane potential (Figs. 6A and 6B). Therefore, to explore the synergistic action of the combination treatment, we focus on the effect of the combination treatment with low-dose (2 and 4 Gy) IR and DHA.

Our findings that combination treatment with DHA and low-dose (2 or 4 Gy) IR augments the induction of G2/M cell cycle arrest at 24 h (Fig. 5B) and apoptosis at 36 h (Fig. 5C) in a synergistic manner suggest that the combination therapy tend to effectively improve the cytotoxic action of DHA. These findings strongly corroborate recent *in vitro* data on increased efficacy of DHA in Molt-4, glioma and Jurkat T-lymphoma cells when combined with IR [20], [22]. Many kinds of tumor cells have been found to be resistant to IR, hence requiring increased IR dose for cancer treatment, which leads to higher side effects [7]. Our observations that the G2/M arrest induced by treatment with low-dose (2 and 4 Gy) IR for 24 h disappeared at 36 h after treatment (Fig. 5A) indicate that low-dose IR does not induce serious side effects. More interestingly, low-dose IR synergistically potentiates the cytotoxic action of DHA (Figs. 5C, 6D and 6E), reducing the side effects of IR treatment and enhancing the efficacies of both DHA and IR. These data provide a guideline to the clinical works for cancer therapy by combining DHA and low-dose IR.

In contrast to the majority of previous reports, it is the extrinsic but not intrinsic apoptosis pathway that mediates the synergistic action of the combination treatment with DHA and IR. Recently, numerous studies have shown that stimuli such as gemcitabine or IR enhance the effects of DHA by intrinsic apoptosis pathway, participated by Bcl-2 family members, p53, c-myc, and NF-κB [15], [17]. Our data show that NAC pretreatment remarkably prevents DHA-induced activation of capase-8 (Fig. 3E) and loss of Δψ_m_ (Fig. 3F), demonstrating that ROS elicited rapidly from DHA triggers both extrinsic and intrinsic pathways to mediate DHA-induced apoptosis. Although IR treatment remarkably enhances DHA-elicited ROS production (Fig. 2C), the fact that IR treatment does not enhance DHA-induced loss of Δψ_m_ (Figs. 6A and 6B) and activation of caspase-9 (Fig. 6C) demonstrate that the intrinsic apoptosis pathway is not involved in the synergistic action of the combination treatment. In combination with the remarkable synergistic efficacy of the combination treatment in apoptosis, our findings that IR treatment significantly accelerates DHA-induced activation of caspase-8 (Fig. 6D) and -3 (Fig. 6E) suggest that the extrinsic apoptosis pathway may play a key role in the synergistic action of the combination treatment.

However, the detailed mechanisms by which low-dose IR synergistically enhance DHA-induced apoptosis via a caspase-8-dependent pathway is unclear. Low-dose IR induces a long lasting production of ROS (Figs. 2A and 2B) which induce G_2_/M cell cycle arrest but not apoptosis (Fig. 5). It is generally considered that the cellular effects of low-dose IR identify mitochondria as the direct target for the generation of ROS [23]. However, our data show that IR neither induces apoptosis (Figs. 5A and 5C) nor enhances DHA-induced intrinsic apoptosis pathway (Figs. 6A, 6B and 6C), indicating that ROS induced by low-dose IR is not generated from mitochondria. More interestingly, low-dose IR remarkably enhances DHA-elicited ROS generation (Figs. 2A and 2B), but does not potentiate DHA-triggered intrinsic apoptosis pathway (Fig. 6). Low-dose IR may act as a catalyzer to break the endoperoxide bridge contained in DHA. However, it is unreasonable to infer that increasing ROS from DHA by IR potentiates the extrinsic but not intrinsic apoptosis pathway due to the fact that DHA activates ROS-dependent extrinsic and intrinsic apoptosis pathways (Fig. 3). The notion that IR is a well-known physical factor that can generate ·OH radicals due to radiolysis of water molecules [22], [24] provides an explanation. IR induces the photolysis of substances such as water molecules to generate ROS that lessen the activation threshold of caspase-8. Another explanation is that the cells arrested in G2/M phase by low-dose IR are more caspase-8-sensitive to DHA.

Although the extrinsic and intrinsic pathways play important roles in DHA-induced apoptosis (Fig. 3), our observations show that inhibitory effects of zIETD-fmk on the cytotoxicity of DHA are much larger than zLEHD-fmk at both 24 and 48 h after DHA treatment (Fig. 3C), demonstrating that the extrinsic pathway plays a more important role in DHA-induced apoptosis. Moreover, this assertion is further demonstrated by our finding that inhibitory effects of zIETD-fmk on DHA-induced caspase-3 activation are far higher than that of zLEHD-fmk (Fig. 3D). In addition, the fact that inhibition of caspase-8 significantly prevents DHA-induced loss of Δψ_m_ (Fig. 3F) and activation of caspase-9 (Fig. 3G) further demonstrates the important role of caspase-8 in DHA-induced apoptosis of A549 cells.

In conclusion, low-dose IR remarkably enhances DHA-induced ROS generation and G_2_/M arrest as well as apoptosis in a synergistic manner via a ROS-mediated extrinsic apoptosis pathway, which provides a potential therapeutic strategy for the clinical treatment of human lung adenocarcinoma.

## Materials and Methods

### Reagents

DHA was obtained from Bide Pharmaceutical Corporation (Guangzhou, Guangdong Province, China). Working solutions were prepared by dissolving the compound in dimethyl sulphoxide (DMSO) before experiments. The final concentration of DMSO was less than 1% in all experiments. N-acetyl cysteine (NAC), Hoechst 33258, RNase A and propidium iodide (PI) were obtained from Sigma (St.Louis, USA). Mouse monoclonal anti-Bcl-x_L_ and anti-β-actin antibodies were obtained from Cell Signaling (Beverly, Massachusetts). All the secondary antibodies were supplied by Molecular Probes (Eugene, Oregon).

### Cell Culture and Transfection

A549 cell line obtained from the Department of Medicine, Jinan University (Guangzhou, China) was cultured in Dulbecco’s modified Eagle’s medium (DMEM, Gibco, Grand Island, USA) supplemented with 10% fetal calf serum. Cell cultures were maintained at 37°C in a humidified 5% CO_2_ incubator. For fluorescence studies, cells were transiently transfected with plasmids using Turbofect™ *in vitro* transfection reagent (Ferments, USA).

### RNA Interference (RNAi)

The Bax/Bak/Mcl-1 suppression was accomplished using Bax/Bak/Mcl-1 shRNA constructs as described previously [12]. The oligonucleotides for shRNA were synthesized as follows. shBax: 5′-GGGACGAACTGGACAGTAACATTCAAGAGATGTTACTGTCCAGTTCGTC CCTT-3′. shBak: 5′-GCCTGTTTGAGAGTGGCATCATTCAAGAGATGATGCCACTCTCAAACA GGCTT-3′. shNC: 5′-GTTCTCCGAACGTGTCACGTCAAGAGATTACGTGACACGTTCGGAGA ATT-3′. The shRNA sequences were transfected into cells using Turbofect™ siRNA transfection reagent (Ferments, USA) according to the manufacturer’s protocol.

### Cell Viability and Apoptosis Assay

Cell viability was assessed by Cell Counting Kit-8 (CCK-8, Dojindo, Japan) assay as described previously [25], [26]. All experiments were performed in quadruple occasions. Morphological examination for apoptosis was detected by Hoechst 33258 staining. The images of Hoechst 33258 were recorded using a digital camera (Nikon, Tokyo, Japan) with 1280×1280 pixels resolution. Cell apoptosis detection was also performed by flow cytometry (FCM) analysis using Annexin V-FITC/PI apoptosis detection kit (Bender Medsystems, Vienna, Austria) as previously described, and for each FCM analysis 10,000 events were recorded.

### Measurement of Mitochondrial Membrane Potential (Δψ_m_)

Rhodamine 123 (Rho 123, Sigma, St.Louis, USA) was used to analyze Δψ_m_ by flow cytometric (FCM) assay as previously described [18]. Briefly, cells were harvested and stained with 10 µM Rho 123 for 30 min at 37°C in the dark, and then washed with PBS twice and subsequently assayed by FCM. Results were expressed as the proportion of cells with low Rho123 fluorescence indicating the loss of Δψ_m_.

### Cell Cycle Analysis

The proportions of cells in Sub-G_1_ (apoptosis), G_0_/G_1_, G_2_/M, and S phases were determined by FCM analysis of DNA content as described previously [18]. To evaluate the cell cycle profile, cells (about 1×10^6^ cells) were harvested, washed twice with PBS and fixed in ice-cold 70% (v/v) ethanol for 1 h at 4°C. Prior to analysis, samples were washed again and incubated in PBS containing 10 µg/ml RNase A for 30 min, and then incubated with 5 µg/ml PI at 37°C in the dark for 30 min. DNA content was determined using a FCM (FACS, Arla BD, San Jose, California), and data were analyzed by ModFit LT software. For each analysis, 30,000 events were recorded.

### Confocal Microscopy Imaging of Bid and Bax Distribution Inside Living Cells

Fluorescence imaging was performed on a confocal microscope (LSM510/ConfoCor2, Zeiss, Jena, Germany). For the imaging of CFP-Bid, GFP-Bax and DsRed-mito, CFP was excited with 458 nm laser and emission was recorded through a 470–500 nm band pass filter; GFP was excited with 488 nm laser and emission was recorded through a 500–550 nm band pass filter; DsRed was excited with 543 nm laser and emission was recorded through a 565–615 nm long pass filter.

### FCM Analysis of the Activation of Bak and Bax

Cells were grown in 6-well dishes and treated with different conditions, then were harvested and fixed with 4% formaldehyde in PBS for 10 min at 37°C. Afterwards, the cells were treated with ice-cold 100% methanol for 10 min to permeabilize the cells. Fixed cells were blocked in PBS solution containing 1% Bovine serum for 10 min at room temperature and then were incubated with either anti-Bax (6A7) or anti-Bak (Ab-2) (1∶50) at room temperature for 60 min and then incubated with FITC-conjugated goat1anti1mouse IgG (1∶200) for 30 min in the dark. After washing, the samples were analyzed by FCM. The results for each condition were calibrated by values for cells stained with mouse IgG as the primary antibody. Values for untreated controls were arbitrarily set to 100%. In parallel, cells for each condition were stained with antibodies to total Bax or Bak for comparison as described previously [12].

### Fluorometric Determination of Caspase Enzymatic Activation

Activities of caspase-3, -8, and -9 were measured using Ac-DEVD-AFC, Ac-IETD-AFC and Ac-LEHD-AFC (Alexis, Switzerland), respectively, according to the manufacturer’s instructions [11]. Collected cells were washed twice with cold PBS, and were lysed in lysis buffer (50 mM Tris-HCl, pH 8.0, 150 mM NaCl, 1% Triton-100, 1 mM PMSF and protease inhibitor cocktail set I). The extract was transferred to a microlon ELISA plate with 98.5 µl/well. Proluminescence caspase-3, -8, or -9 substrates were added to extract in each well of a microtiter ELISA plate at a 100 µM final concentration at room temperature. Caspases activity was measured continuously by monitoring the release of fluorogenic AFC at 37°C. In the presence of caspase-3, -8, or -9, aminoluciferin was liberated from the proluminescence substance and utilized as a substrate for the luciferase reaction. The resultant luminescence in relative light units was measured by using auto microplate reader (infinite M200, Tecan, Austria). The excitation wavelength of AFC was 405 nm and the emission detection channel was 478–535 nm. The reaction mixture without protein was referred to the background and was subtracted from samples, and the caspase activation level in control cells was normalized to 1.0.

### DHA and IR (X-ray) Treatment

Cells were treated with 20 µg/mL of DHA for different time without specially explanation. IR (X-ray) was done at room temperature with 6 MV photons from a linear accelerator (Siemens, Germany) at a dose rate of 6 to 15 Gy/min. For combined treatment, DHA was added 30 min before IR treatment. Pretreatment with the caspase-8 and -9 as well as ROS inhibitors zIETD-fmk, zLEHD-fmk (San Francisco, USA) and NAC were done 1 h before further treatment, respectively.

### Measurement of Intracellular ROS Generation

ROS generation inside living cells was measured by FCM with 2′,7′-Dichlorodihydrofluorescein diacetate (DCFH-DA) (Wako Ltd, Osaka, Japan), an oxidation-sensitive probe, which is cleaved by nonspecific esterases and turns to highly fluorescent DCF upon oxidation by ROS. Untreated or treated cells were stained with 20 µM DCFH-DA for 30 min in the dark and subsequently assayed by FCM.

### Western Blotting Analysis

Cells were lysed in lysis buffer (50 mM Tris-HCl, pH 8.0, 150 mM NaCl, 1% Triton-100, 1 mM PMSF and protease inhibitor cocktail set I). After removing insoluble material by centrifugation for 5 min at 12,000×g, the protein concentration was estimated in the supernatant using the Bio-Rad protein assay (Bio-Rad, Munich, Germany) according to the manufacturer’s protocol. Protein was separated by SDS-PAGE under reducing conditions before transferring onto nitrocellulose membranes (Millipore, Billerica, USA). Blots were blocked in TBST buffer containing 5% non-fat dry milk for 1 h at room temperature. The membrane was incubated overnight at 4°C with the respective primary antibodies. After repeated washings with TBST, the membranes were incubated with the secondary antibody for 1 h at room temperature before continuing to wash with TBST. Detection was performed using the Odyssey Infrared Imaging System (LI-COR Biosciences, Nebraska, USA). Equal loading was verified by antibody against β-Actin.

### Statistical Analysis

Data were presented as means ± SD. Data were analyzed by repeated-measures ANOVA with parametric methods and LSD multiple comparison using the statistical software SPSS 10.0 (SPSS, Chicago). Two-tailed Student’s t-test was also performed where appropriate. Throughout the work, *P* values less than 0.05 were considered to be statistically significant. All experiments were performed in a minimum of three times.
